# Hearing shapes: the Bouba–Kiki effect in adult domestic dogs

**DOI:** 10.1098/rspb.2026.1209

**Published:** 2026-10-14

**Authors:** Eleonora Biffi, Giulia Pedretti, Simona Normando, Teddy Lazebnik, Maria Loconsole, Paola Valsecchi

**Affiliations:** ^1^ Department of Medicine and Surgery, University of Parma, Parma 43126, Italy; ^2^ Department of Chemistry, Life Science and Environmental Sustainability, University of Parma, Parma 43124, Italy; ^3^ Department of Comparative Biomedicine and Food Science, University of Padova, Legnaro 35020, Italy; ^4^ Department of General Psychology, University of Padova, Padova 35131, Italy; ^5^ Department of Information Systems, University of Haifa Department of Israel Studies, Haifa 3498838, Israel; ^6^ Department of Computing, Jönköping University, Jönköping 551 11, Sweden

**Keywords:** sound symbolism, Bouba–Kiki effect, domestic dog, cross-modal correspondence

## Abstract

The ‘Bouba–Kiki effect’ is an example of spontaneous association between auditory and visual stimuli, described in humans as a case of sound symbolism, a predisposition linked to language. Recent evidence showed that 3-day-old chicks also exhibit a spontaneous association between round and spiky shapes and the sounds ‘Bouba’ and ‘Kiki,’ respectively, thus suggesting that the effect may rather be an instance of a more widespread cross-modal correspondence. We investigated whether the Bouba–Kiki effect is present in adult domestic dogs (*Canis familiaris*) by investigating dogs’ spontaneous tendency to associate a spiky or a rounded shape to the ‘Bouba’ and ‘Kiki’ sounds. Dogs looked significantly more at the rounded shape while ‘Bouba’ was played, and they looked at and explored the spiky shape when ‘Kiki’ was played. Our results suggest that a spontaneous sound–shape association akin to that of humans is also present in adult domestic dogs. Further studies are needed to investigate the function and ontogenesis of this cognitive process.

## 1. Introduction

The term sound symbolism refers to the association between sounds and specific perceptual elements. Interest in this topic has grown within psychology, with studies demonstrating that sound-symbolic effects broadly influence humans, not only in language development [1], but also in behaviour and cognition [2,3]. Perhaps the most well known example of sound symbolism is the Bouba–Kiki effect [4]. This effect describes the tendency to associate the pseudowords ‘Bouba’ with a rounded shape, and ‘Kiki’ with a spiky shape [5]. This spontaneous matching between shapes and sounds has been repeatedly demonstrated through explicit matching tasks and implicit association tests [6]. Moreover, it has been observed in adults from diverse linguistic and orthographic backgrounds [7,8], as well as in preverbal infants [1], suggesting that it is a widespread effect, present at the early stages of development. However, studies investigating the Bouba–Kiki effect in non-human animals are marked by mixed outcomes. Attempts to demonstrate the effect in non-human primates, such as chimpanzees and gorillas, were unsuccessful [9], reinforcing the view that this is in fact a phenomenon unique to our species. At the same time, there is evidence that 3-day-old chicks (*Gallus gallus*) exhibit a spontaneous association between rounded and spiky shapes and the sounds ‘Bouba’ and ‘Kiki’ [10]. This latter study points towards the idea that, rather than being a language-specific phenomenon related to sound symbolism, the Bouba–Kiki effect may be an instance of systematic mapping between sensory attributes across modalities, namely cross-modal correspondences [5]. Cross-modal correspondences are shared across different species, such as chimpanzees [11], monkeys [12], dogs [13,14], chickens [10,15] and tortoises [16,17]. They are thought to have ecological significance, as they could enable individuals to predict association based on the likelihood of two dimensions correlating in nature [8]. For instance, in the case of pitch–size correspondence [13,16], this probably reflects the fact that, owing to physical constraints of the vocal tract, small animals produce higher-pitched sounds than large animals, whereas larger animals produce lower-pitched sounds [18,19]. A similar explanation, based on statistical regularities and the co-occurrence of sensory information, may underlie the Bouba–Kiki effect. Round objects, when hitting or rolling on a surface, typically produce lower-frequency, more continuous sounds compared with spiky objects of the same size [20].

In this study, we investigate the Bouba–Kiki effect in adult domestic dogs (*Canis familiaris*), aiming to provide novel evidence from non-human mammals and deepen our understanding of the function of sound–shape associations. As domestic dogs process and respond to human language using mechanisms analogous to humans, they constitute a promising comparative model for the study of sound symbolism. A minority of domestic dogs can learn to associate a new word with a new object after a single exposure, like human infants do (fast mapping) [21,22]. More recently, it has been shown that a small number of dogs (named ‘Gifted Word Learner’) are able to learn objects’ labels also from overheard speech, a socio-cognitive process that is present in both adult and infant humans [23]. Thus, demonstrating the Bouba–Kiki effect in domestic dogs would not only provide novel evidence for its presence in a non-human species, but also lay the groundwork for future studies aimed at deepening our understanding of the mechanisms underlying it. To this end, we presented dogs with visual shapes (either rounded or spiky) projected on two screens and paired with the sounds ‘Bouba’ or ‘Kiki’. We examined their spontaneous looking preference through an artificial intelligence (AI) computational model that analysed dogs’ angular vision and exploratory behaviour through manual coding. The preferential looking paradigm has been widely used to examine visual attention and the correspondence between visual and auditory stimuli not only in humans (e.g. infants [24–27]), but also in several non-human species, including chicks [10], rhesus monkeys [28,29], horses [30,31], tufted capuchin monkeys [32], bonobos [33] and domestic dogs [19,34–36]. This paradigm assumes that looking time is a valid measure of an individual's interest and perceptual or cognitive processing of a given stimulus [37]. We predicted that if dogs, like humans and chicks, respond to the Bouba–Kiki effect, they will (1) preferentially look at the round shape paired with the ‘Bouba’ sound, and the spiky shape paired with the ‘Kiki’ sound; and (2) engage in more exploratory behaviours towards the shape they spontaneously matched with the auditory stimulus.

We tested 51 adult domestic dogs (see supplementary material, table S1) with the owner able to see the shapes and listen to the sounds (Study 1). To rule out potential biases owing to the so-called Clever Hans effect, namely unintentional owner signals influencing the dogs’ responses, we tested an additional group of 19 subjects whose owners wore headphones playing music to prevent them from hearing the test sounds (see supplementary material, table S2) (Study 2). In both studies, each dog was tested in four consecutive trials, each lasting 30 s. The order of ‘Bouba’ and ‘Kiki’ trials was counterbalanced between subjects.

## 2. Material and methods

### (a) Subjects

#### (i) Study 1

We tested 51 mesocephalic medium- to large-sized domestic dogs: 27 males (intact = 17 and neutered = 10) and 24 females (intact = 9 and neutered = 15) with an age between 1 and 12 years (mean = 6.3 years) (see supplementary material, table S1 for more details). Dogs were recruited through the University of Parma database and social media platforms.

#### (ii) Study 2

Based on a *post hoc* power analysis (power = 0.858), we tested 19 additional mesocephalic dogs: 8 males (intact = 6 and neutered = 2) and 11 females (intact = 5 and neutered = 6) with an age between 1 and 11 years (mean = 5.28 years) (see supplementary material, table S2 for more details). Subjects were recruited through dog training centres.

### (b) Experimental design and setup

Studies 1 and 2 were identical in all the experimental procedures, with the only exception being that in Study 2 the owners were unaware of the auditory stimuli presented to the dogs, as they were wearing headphones playing rock music.

The experiments were conducted in the Dog's Ethology Lab of the University of Parma, Italy. The experiment was conducted in an indoor room (3 × 4 m), which was divided into two sections by a wooden panel (figure 1). The wooden panel featured two openings, one on the right and one on the left, where two identical screens (27 inch E2723H, DELL, TX, USA) were positioned to display the visual stimuli (Bouba shape or Kiki shape [8], figure 1). Two speakers (JBL Flip 6 Bluetooth speakers) were placed behind each screen to deliver the auditory stimuli (‘Bouba’ sound or ‘Kiki’ sound [8]). Behind the apparatus, there was a designated area for the experimenter, who operated a computer connected to the screens, speakers and a GoPro 10, placed on top of the wooden panel for video recording. In front of the apparatus, at 3 m, a chair for the dog's owner was positioned.

**Figure 1.**
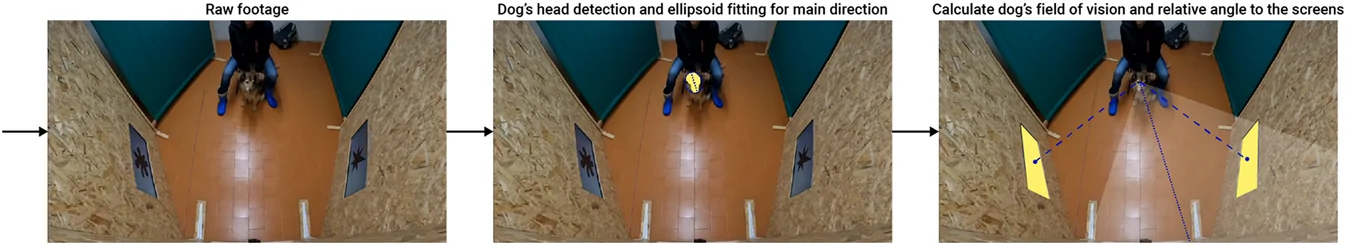
Setup of the experiment with the two screens, and steps of the computational analyses with dog's head detection and angular vision detection.

### (c) Experimental stimuli and procedure

The experiment was run with a dedicated Python program, which synchronized the auditory and visual stimuli presentation along with video recording.

The visual stimuli consisted of a black spiky shape and a black rounded shape (figure 2) based on previous studies [7,8,10], which demonstrated the cross-modal association Bouba–Kiki across various cultures and writing systems. The auditory stimuli comprised repeated pronunciations of ‘Bouba’ and ‘Kiki’; these sounds were originally spoken by the first author of a previous study [8], a trained female Polish phonetician. Each auditory stimulus was presented 15 times over a 30 s period per trial. Figure 3, in which the frequency (Hz) is plotted as a function of time (s) to represent energy distribution (dB), highlights the distinctive acoustic properties corresponding to the pseudowords ‘Kiki’ and ‘Bouba.’ The ‘Kiki’ spectrogram is characterized by a high-frequency spectral profile, whereas the ‘Bouba’ spectrogram exhibits a low-frequency spectral profile.

**Figure 2.**
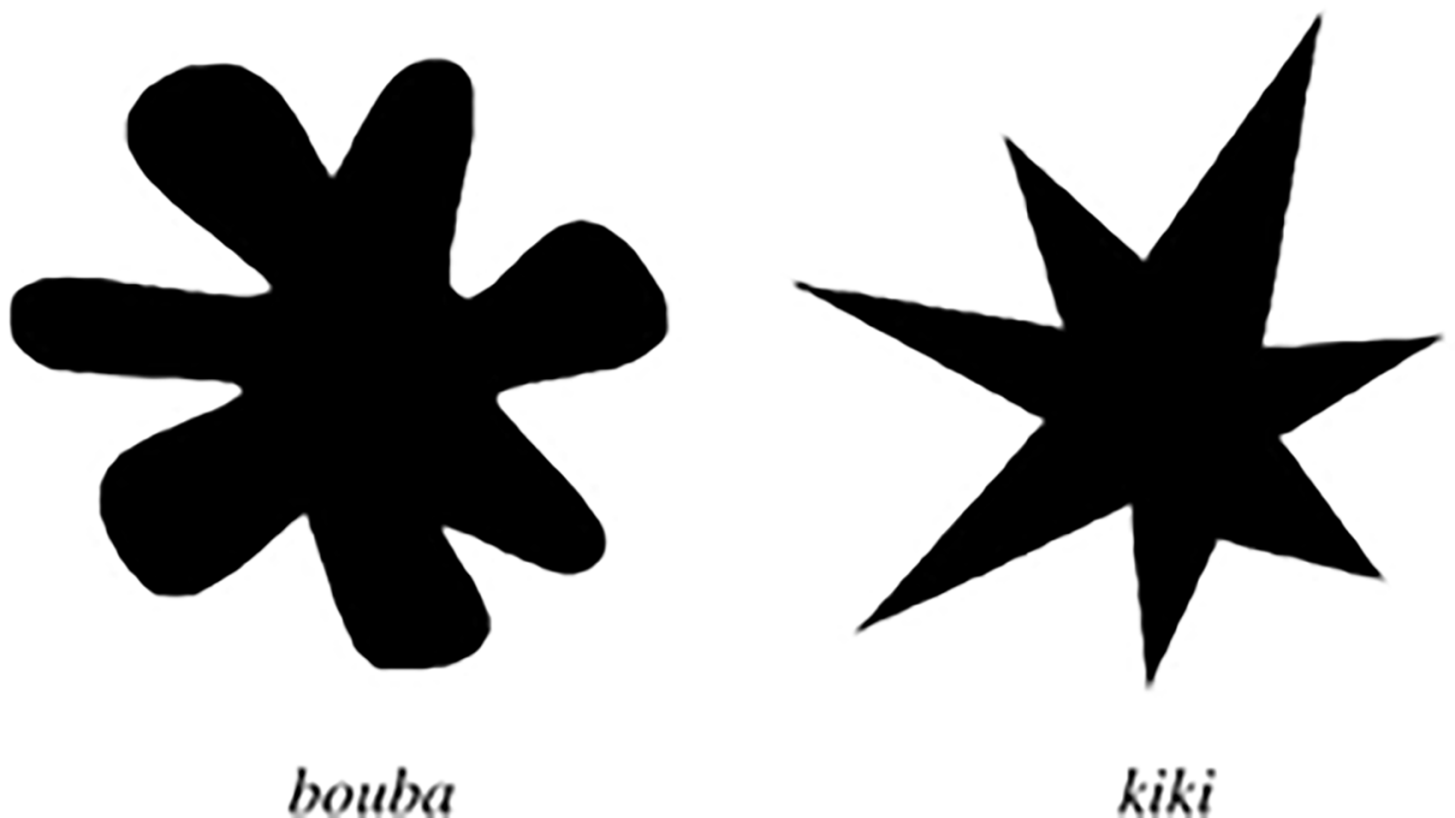
Visual stimuli representing the rounded shape (on the left) and the spiky shape (on the right).

**Figure 3.**
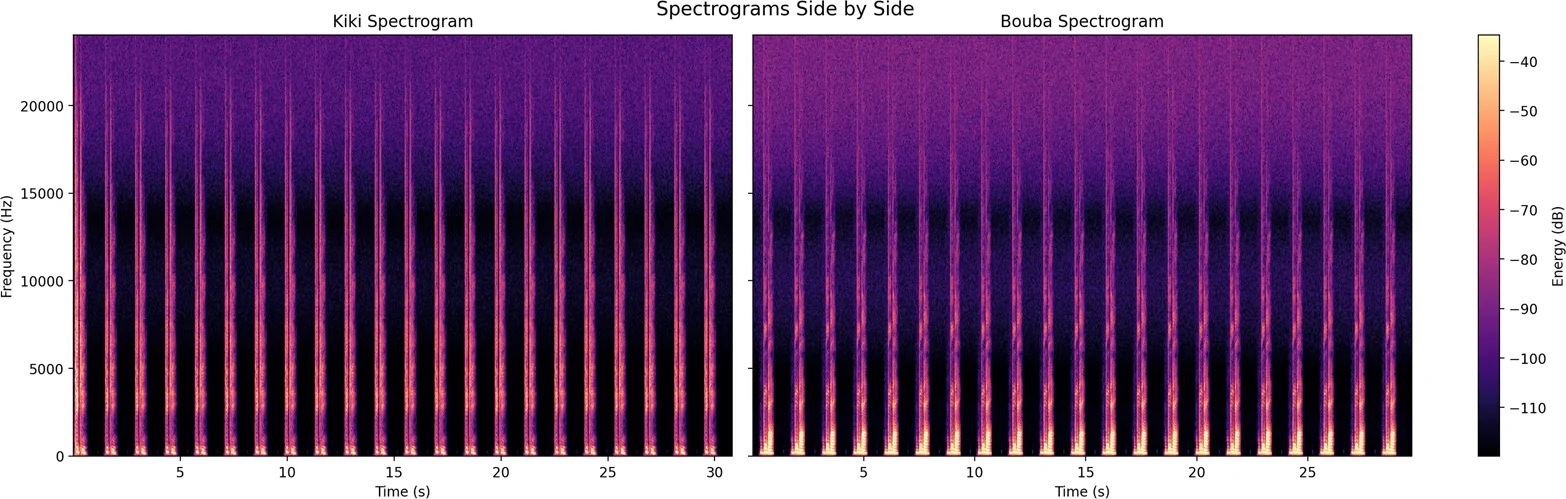
Comparative spectrogram, with the frequency (Hz) plotted as a function of time (s) to represent energy distribution (dB), of the pseudowords ‘Kiki’ and ‘Bouba’.

Before the experiment, the owner filled out a consent form. Upon arrival at the laboratory, the dog was allowed to freely explore the room for 3 min, to get comfortable with the apparatus and the environment. Before the dog entered, a total of six pieces of food rewards were placed on the ground near the apparatus, two in front of each screen and two in the centre part of the apparatus. During the exploration, the experimenter explained the procedure to the dog's owner. Following the exploration phase, the owners sat and called the dog near them. The dog was held still by the owner with its shoulders aligned towards the middle of the apparatus, ensuring the dog was positioned straight, facing the centre of the apparatus. In Study 1, the owner was only instructed to look straight towards the middle of the apparatus; instead, in Study 2, the owner wore headphones in order not to hear the sound played (‘Bouba’ or ‘Kiki’). The study followed a within-subject design where each dog underwent four identical test trials in one session lasting about 15 min. The order of auditory stimuli (‘Bouba’ or ‘Kiki’) was counterbalanced and alternated across the four trials across dogs, and the spatial position (left or right) of the visual stimuli alternated.

The possible combinations of sound and visual stimuli are reported below:

(i) BOUBA_bk: ‘Bouba’ sound with the Bouba shape on the right screen and the Kiki shape on the left screen;
(ii) BOUBA_kb: ‘Bouba’ sound with the Kiki shape on the right screen and the Bouba shape on the left screen;
(iii) KIKI_kb: ‘Kiki’ sound with the Kiki shape on the right screen and the Bouba shape on the left screen;
(iv) KIKI_bk: ‘Kiki’ sound with the Bouba shape on the right screen and the Kiki shape on the left screen.

Each test session started with either the ‘Bouba’ or ‘Kiki’ sound paired with one of the two possible image positionings (‘bk’ or ‘kb’). The subsequent trial featured the other auditory stimulus. The order of the eight combinations was counterbalanced across subjects (supplementary material, tables S1–S3).

During each trial, the auditory stimulus (either ‘Bouba’ or ‘Kiki’) was played for 30 s, with a total of 15 repetitions, while the visual stimuli were shown simultaneously.

The trial was divided into two phases:

(i) Spontaneous looking phase (20 s): the shapes were outlined with a red frame, and the dog was held in position.
(ii) Exploratory phase (10 s): the frame turned green, signalling the owner to release the dog, allowing it to explore the stimuli freely.

A 1 min break followed each trial, during which the dog was allowed to explore the room. At the end of the break, the owner recalled the dog and positioned it for the next trial. The same procedure was repeated for all four trials.

### (d) Behavioural coding

The first look towards one of the two screens and its latency were calculated through the AI model. The behaviour of spontaneous looking of the dogs in the first 20 s of each trial session was analysed by an AI model (see §2e). The duration (s) of looking at the screens was calculated by the AI model (see §2e). The AI model was able to process and analyse over 91% of the recorded frames. This performance is comparable with other animal analysis studies; thus the AI model proved to be an effective and reliable method [38,39].

The exploratory behaviour of the last 10 s of each trial (‘exploring bouba’, ‘exploring kiki’, ‘no exploration’—see supplementary material, table S4) was coded manually through BORIS (Behavioral Observation Research Interactive Software [40]) by one coder (E.B.). An external behaviour coder also analysed 50 videos for the inter-rater reliability with the main coder; intra-class correlations (ICCs) (RStudio v. 2024.09.1) revealed good reliability—ICC between 0.883 and 0.937 (*p*-value < 0.001).

### (e) Computational analyses of angular vision

In the first 20 s, the preferential looking towards the screen was evaluated to define an association between the shape and the sound. An AI model was developed to analyse videos of dogs to determine their gaze direction, a critical metric for studying attention and decision-making processes. The proposed model's workflow began by standardizing video data, ensuring consistency in temporal resolution by sampling frames at 30 frames per second. This standardization was necessary to eliminate inconsistencies arising from varied video frame rates, which could introduce noise into behavioural analyses. Each frame was then processed to detect the dog's head orientation, as it serves as a proxy for gaze direction. For this purpose, we employed the YOLOv8 model, a state-of-the-art object detection algorithm optimized to locate the head efficiently, even under varying lighting conditions and head orientations. The YOLOv8 model [41] was fine-tuned on a custom dataset of 280 images, each manually annotated with bounding boxes around the dogs’ heads. The images were taken at random from the set of frames captured in our experiments. These annotations were carefully curated to provide a robust foundation for detecting head positions across diverse scenarios. After identifying the head's position, the model applied the Canny algorithm [42] to detect its edge. The edge delineated the border of the head, which was then used to generate a segmentation mask—representing the head's interior region. This segmentation was further analysed to fit an ellipsoid around the head. We used the ellipsoid, and not squares or circles, because it has the main and secondary axes (not present in squares and circles) useful to determine the direction of the dog's head. The ellipsoid fitting [43] process was driven by a two-dimensional Gaussian distribution applied to the segmented pixels, ensuring that the ellipsoid's axes aligned with the head's natural orientation. Simultaneously, the model identified screens within the frame, representing the visual stimuli of interest. These screens were detected by searching for blocks of pixels within a specified RGB (red, green and blue) colour range, defined between (220, 220, 220) and (255, 255, 255) of at least 500 pixels. This colour range and number of pixels were chosen to reliably isolate screens from other elements in the frame. The proposed model assumed the presence of two screens and extracted their bounding trapezoids to define their spatial extents. The final step involved computing the angle between the major axis of the ellipsoid (which reflects the head's orientation) and the line connecting the ellipsoid's centre to the centre of each screen's trapezoid. If the angle between these vectors was less than 30°, the frame was classified as the dog being oriented towards the respective screen. This angle threshold was selected based on empirical observations to balance sensitivity and specificity in gaze classification. The time spent looking at the right or left shape was assessed through the estimation of the direction of the head–-nose line and the assessment of the binocular angle of vision. In accordance with Maggs and colleagues [44], we decided to consider only the binocular vision of dogs to determine if they were looking at the screen or not. After computing the binocular angle, we defined the dog to be looking at a screen if the binocular vision angle included the screen. We noted the number of frames in which the dog had its binocular angle directed towards one of the screens. Based on these assumptions, we considered the frames in which the dog was looking at a screen to be those where there is an intersection between the dog's gaze and at least one of the screens.

### (f) Statistical analysis

From the computational analyses, we extracted the first look towards one of the two screens, latency from the start of the trial to the first look, and the number of frames in which the binocular angle was directed to one or the other screen. From the behavioural coding, we calculated the occurrence and duration of the exploratory behaviour towards the spiky or rounded shape in the 10 s of exploration.

To control for a potential general side bias, the mean looking proportion towards the right and left sides of the apparatus was analysed using a one-sample *t*-test [45]. To check for a preference towards one of the two shapes independently from the auditory stimuli, we analysed: (i) the direction of the first look with a *χ*^2^-test [46]; (ii) the first look latency using a generalized linear mixed-effects model (GLMM) with a gamma distribution and log link, including shape as a fixed effect and subject as random intercept, fitted with the ‘glmmTMB’ package [47]; (iii) the overall time spent looking at the two shapes using a GLMM with a beta distribution (function ‘glmmTMB’, package ‘glmmTMB’ [47]).

To investigate whether the dogs’ gaze, in the first 20 s, was oriented at the spiky or rounded shape when the ‘Kiki’ or the ‘Bouba’ sound was played, a GLMM with a beta distribution (function ‘glmmTMB’, package ‘glmmTMB’ [47]) was used. A beta regression was used since the response variable considered was the proportion of frames in which the dog was looking at the spiky or rounded shape divided by the total number of frames in which the dog was looking at the screens (proportion bounded between 0 and 1). The sound (two-level factor ‘Kiki’/‘Bouba’) was included as the main predictor. Trial, sex and age were included as control predictors, and the subject ID as a random effect to control for repeated observations of the same individual.

A GLMM was also used to find whether the number of trials (test predictor) influenced the duration of the looking time at the screen negatively, with sex and age as control predictors and subject ID as a random effect.

To analyse the exploration pattern of the dogs during the last 10 s of each trial, firstly exploration values greater than 10 s were set to 10 s, then a GLMM with binomial distribution was used (function ‘glmmTMB’, package ‘glmmTMB’ [47]). Whether the dogs explored the spiky or rounded shape (occurrence, binomial response) was used as a response variable, and the sound was included as the main test predictor, whereas trial, sex and age were included as control predictors. Finally, a GLMM with a gamma distribution was used (function ‘glmmTMB,’ package ‘glmmTMB’ [47]), to evaluate the influence of sound stimuli on the exploratory behaviour towards the screens. As an overall test of the main predictors and to avoid ‘cryptic multiple testing’ [48], we compared the full models as described above with null models lacking the test predictor but otherwise identical. We tested the effect of individual fixed effects using likelihood ratio tests comparing the full models with reduced models lacking the fixed effects one at a time [49]. For these tests as well as the full–null model comparison, we used a likelihood ratio test [50]. Confidence intervals (CIs) for each comparison were calculated with the generic R function ‘confint’. Model stability was estimated, dropping the individuals one at a time from the data and comparing the estimates derived for models fitted to these subsets with those obtained for the full dataset for each model, with the R function ‘glmmTMB.stab’. These revealed all the models to be of good stability (for details see the supplementary material). All models were tested for zero- and one-inflation. No evidence of inflations was detected, which suggests an appropriate chosen model distribution. All statistical analyses were performed in R v. 4.5.1. Full package version information is provided in the the Open Science Framework (OSF) repository. Results were considered statistically significant at *p* ≤ 0.05.

For Studies 1 and 2, the stability of the models was assessed using a function provided by Roger Mundry (Max Planck Institute for Evolutionary Anthropology, Leipzig, Germany), and all of them revealed acceptable stability.

## 3. Results

### (a) Study 1

#### (i) General side bias towards the right or left

One-sample *t*-test revealed no significant side bias towards the right or left screen (for the right side: mean = 0.499, 95% CI [0.490, 0.508], *t*_50_ = −0.24, *p* = 0.81; for the left side: mean = 0.501, 95% CI [0.492, 0.510], *t*_50_ = 0.24, *p* = 0.81).

#### (ii) First look, its latency and overall looking preference regardless of the sound

The dogs’ first look was directed equally towards one of the two shapes ($\chi_{1}^{2}$ = 1.96, *p* = 0.161). A likelihood ratio test revealed a significant effect of shape on the latency responses (full–null model comparison: $\chi_{1}^{2}$ = 4.647, *p* = 0.031), with dogs showing shorter latencies when looking at the spiky shape than the rounded shape (estimate (Es.) −0.18 ± 0.08, *p* = 0.03, 95% CI [−0.35; −0.02]). Dogs looked more towards the spiky shape than the rounded shape (full–null model comparison: *χ*² = 33.764, *p* < 0.001; looking at the spiky shape: Es. 0.27 ± 0.05, *p* < 0.001, 95% CI [0.18; 0.36]).

#### (iii) Looking at the screen

Dogs looked at the screens for an average of 5.8 s (5.8 ± 2.1 s) out of 20 s of trial. No effect of the trial on time spent looking at the screens was found (*p* = 0.24; supplementary material, table S5).

#### (iv) Preferential-looking task

The AI model computed dogs’ binocular vision angle and the proportion of frames in which that angle included the spiky or rounded shape. The proportion of looking frames was influenced by the auditory stimuli played (full–null model comparison: *χ*² = 215.69, *p* < 0.001). Specifically, dogs looked longer at the rounded shape when the ‘Bouba’ sound was played (*p* < 0.001, Es. −0.76 ± 0.04, 95% CI [−0.84, −0.69]), and at the spiky shape when the ‘Kiki’ sound was played (*p* < 0.001, Es. 0.76 ± 0.04, 95% CI [0.69, 0.84]) (figures 4 and 5). No effects of trial, sex, age and order were found.

**Figure 4.**
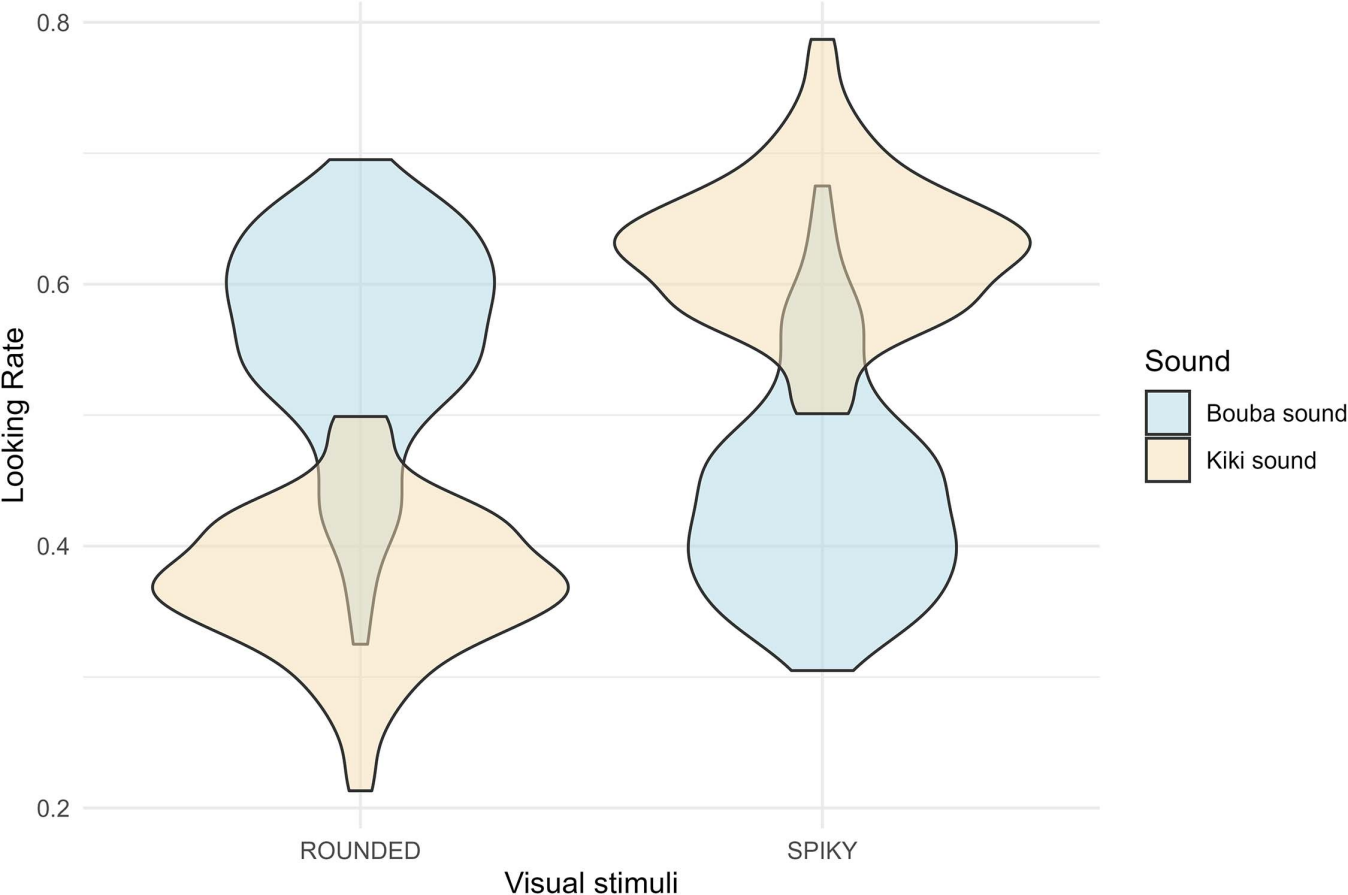
Looking rate of dogs’ binocular vision towards the screen depicting the rounded or spiky shape when the ‘Bouba’ (blue) or ‘Kiki’ (cream) sound was played (Study 1—the owner was able to see the shapes and listen to the sounds).

**Figure 5.**
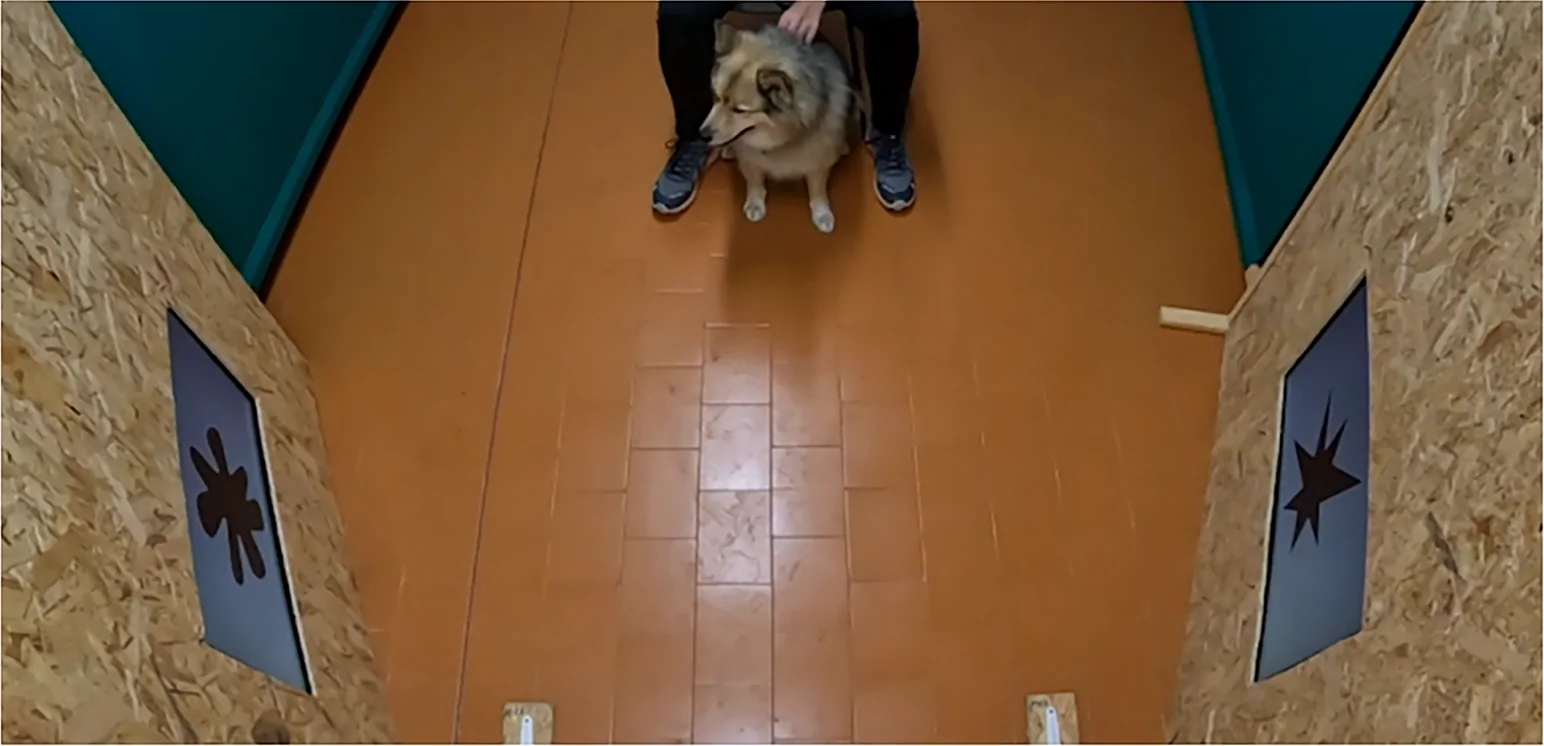
Dog looks at the rounded shape when hearing the ‘Bouba’ sound (Study 1—the owner was able to see the shapes and listen to the sounds).

#### (v) Exploratory behaviours

During the exploratory phase of the trials, when the ‘Kiki’ sound was played, dogs investigated the spiky shape more frequently and for a longer duration compared with the ‘Bouba’ condition (frequency: full–null model comparison: *χ*² = 7.8, *p* = 0.005; Es. 1.07 ± 0.4, *p*-value = 0.007, 95% CI [0.31, 1.89]; duration: full–null model comparison: *χ*² = 14.41, *p* = 0.0001, Es. 1.9 ± 0.6, *p*-value = 0.001, 95% 95% CI [0.89, 3.289]) (figure 6). On the contrary, neither the occurrence nor the duration of exploration of the rounded shape was influenced by the ‘Bouba’ sound played. No effects of trial, sex, age and order were found.

**Figure 6.**
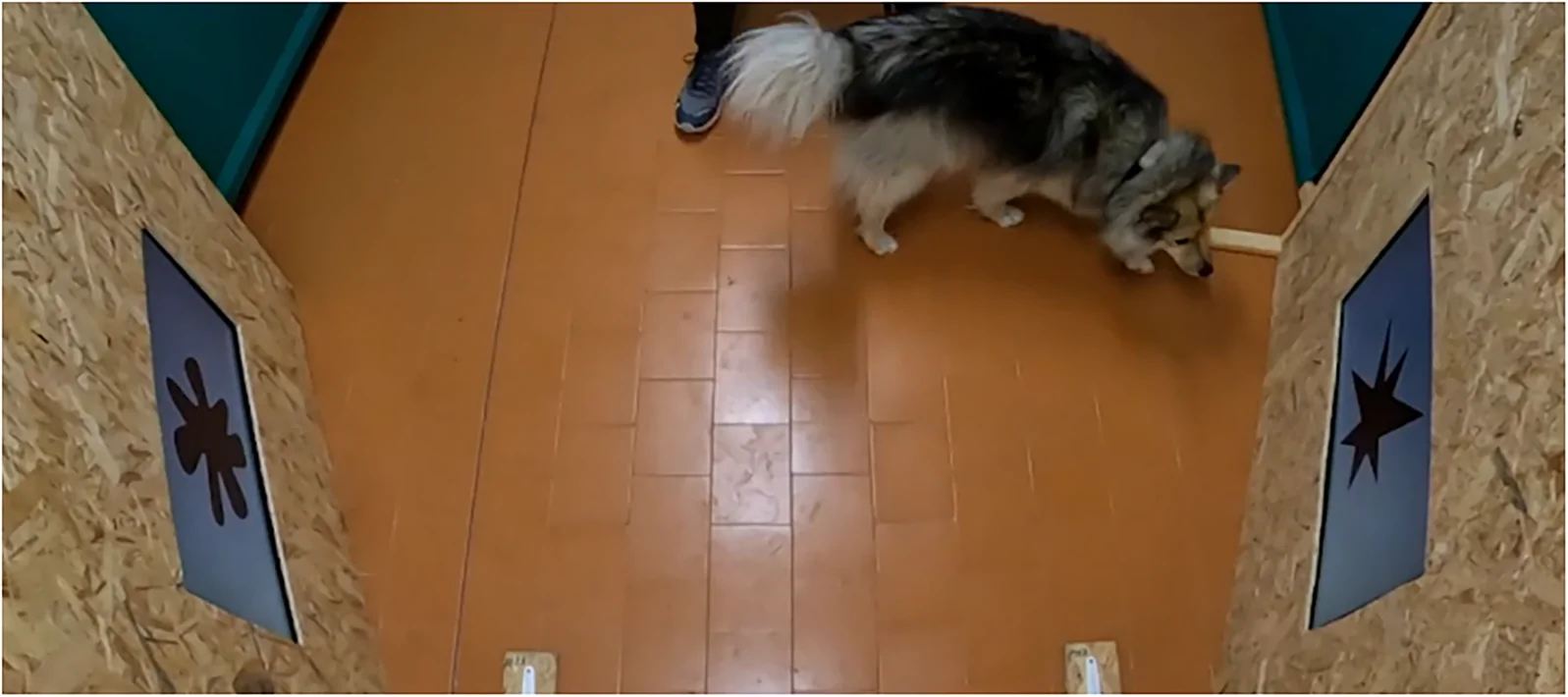
Dog explores the spiky shape when hearing the ‘Kiki’ sound (Study 1—the owner was able to see the shapes and listen to the sounds).

### (b) Study 2

#### (i) General side bias towards the right or left

One-sample *t*-test revealed no significant side bias towards the right or left screen (right mean = 0.498, 95% CI [0.485, 0.511]) and (left mean = 0.502, 95% CI [0.489, 0.515]) (right: *t*(18) = –0.32, *p* = 0.75; left: *t*(18) = 0.32, *p* = 0.75).

#### (ii) First look, its latency and overall looking preference regardless of the sound

The dogs’ first look was directed equally towards one of the two shapes ($\chi_{1}^{2}$ = 0.47, *p* = 0.49). A likelihood ratio test showed no significant difference in the latency responses towards one of the two shapes (full–null model comparison: $\chi_{1}^{2}$ = 1.82, *p* = 0.17). The dogs, in general, did not show an overall looking preference towards one of the two shapes (full–null model comparison: *χ*² = 0.39, *p* = 0.53).

#### (iii) Looking at screens

Dogs looked at the screen for an average of 8.8 s (8.8 ± 1.7 s). No effect of the number of trials on time looking at the screen was found (*p* = 0.4; supplementary material, table S6).

#### (iv) Preferential-looking task

The results of the AI model showed that dogs looked longer at the rounded shape when the ‘Bouba’ sound was played (full–null model comparison: *χ*² = 137.42, *p* < 0.001, Es. −0.97 ± 0.05, 95% CI [−1.07, −0.88]), and at the spiky shape when the ‘Kiki’ sound was played (full–null model comparison: *χ*² = 137.42, *p* < 0.001, Es. 0.97 ± 0.05, 95% CI [0.88, 1.07]) (figure 7).

**Figure 7.**
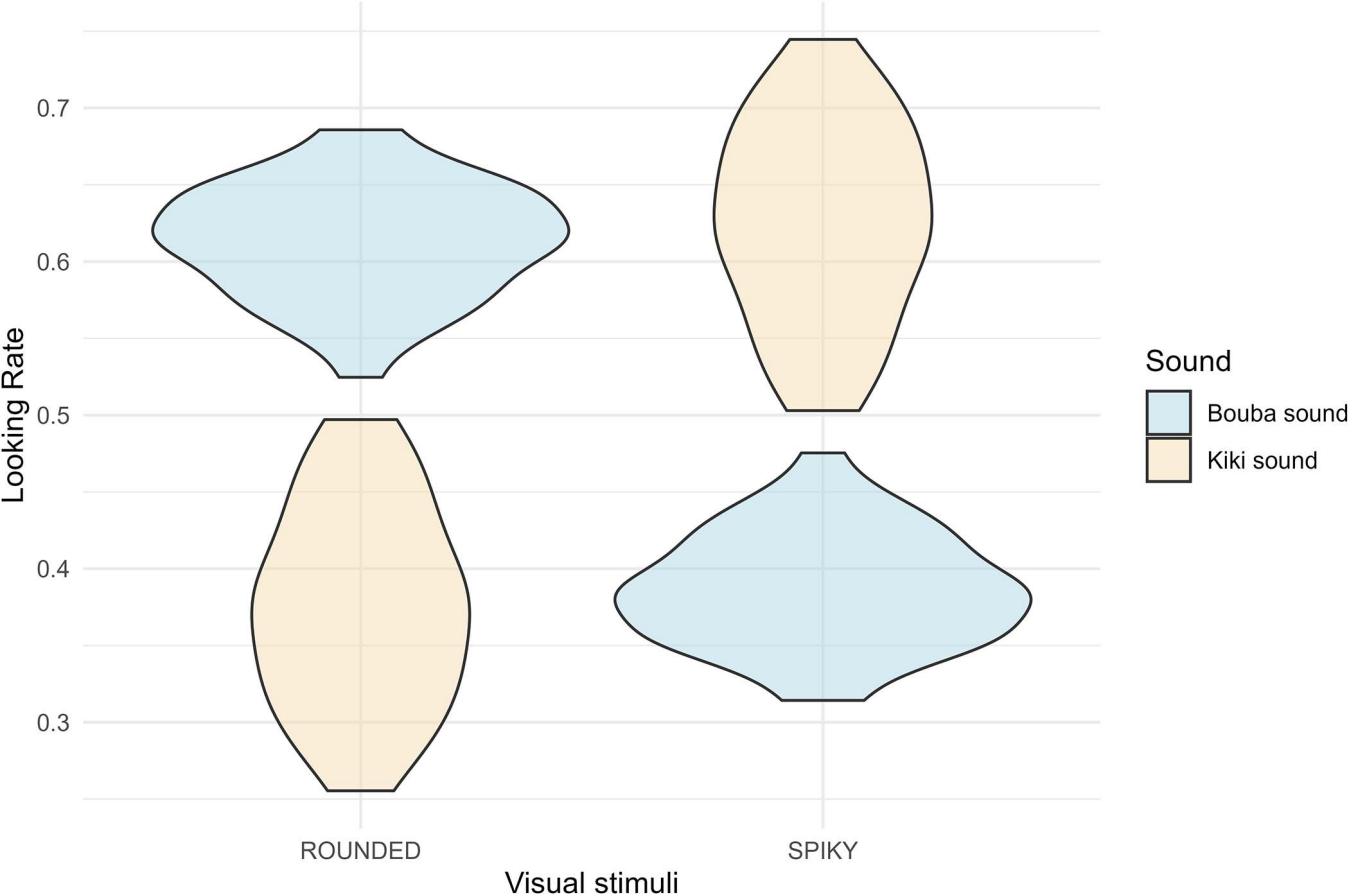
Looking rate of dogs’ binocular vision towards the screen depicting the rounded or spiky shape when the ‘Bouba’ (blue) or ‘Kiki’ (cream) sound was played (Study 2—owner able to see the shapes but not hear the sounds since wearing headphones playing rock music).

#### (v) Exploratory behaviour

Neither the occurrence nor the duration of exploration of the two shapes was influenced by the sounds played (‘Bouba’ sound—occurrence: full–null model comparison: *χ*² = 1.06, *p* = 0.30; duration: full–null model comparison: *χ*² = 1.62, *p* = 0.20; ‘Kiki’ sound—occurrence: full–null model comparison: *χ*² = 0.23, *p* = 0.63; duration: full–null model comparison: *χ*² = 0.6, *p* = 0.45). No effects of sex, age, order and trial were found.

## 4. Discussion

In both Study 1 and Study 2, dogs showed no lateralization towards either the right or the left screen and the first gaze was directed equally towards the two shapes. They also showed longer looking times towards the rounded shape when hearing the ‘Bouba’ sound, and towards the spiky shape when hearing the ‘Kiki’ sound, thus displaying a spontaneous sound–shape association consistent with the Bouba–Kiki effect previously reported in humans and baby chicks. This strengthens the view that the Bouba–Kiki effect is part of a broader set of cross-modal correspondences shared among different species, instead of it being unique to humans and linked to language. In line with this idea, substantial evidence indicates that humans and dogs share several cross-modal correspondences, including pitch–size, pitch–space and pitch–luminance associations [13,14,18]. They can infer another dog's body size from vocal cues—linking high-pitched growls to small dogs and low-pitched growls to large ones [18,19]. Similarly, they pay more attention to audio–visual combinations that follow this pattern, such as high-pitch sounds paired with stimuli located higher [13]. Korzeniowska and colleagues [14] demonstrated that dogs respond faster to congruent pitch–size pairings, showing quicker orientation towards small stimuli emitting high-pitched sounds than towards incongruent combinations.

Interestingly, in Study 1, dogs also displayed: (i) a shorter latency when first looking at the spiky shape, independent of the auditory stimulus; (i) an overall general looking preference for the spiky shape, regardless of the auditory stimuli; (iii) an exploratory preference towards the spiky shape when hearing the ‘Kiki’ sound. Overall, these results may suggest a stronger preference for spikiness over roundness in dogs. This preference may partially explain the results from the exploratory phase, where dogs did not show a shape preference in the ‘Bouba’ condition, but spent longer exploring the spiky shape when hearing ‘Kiki’. This result is in contrast with findings in human infants and in chicks, in which the effect appeared stronger for the rounded–Bouba pairing. The result in baby chicks was interpreted as a byproduct of an innate perceptual preference for roundness, which is advantageous in young animals for maximizing the chances of orienting towards a conspecific (e.g. a face, which has a round outline). We are unaware of any empirical evidence for a comparable shape bias in dogs. As the overall looking preference for spiky shapes emerged in Study 1 but not in Study 2, it could be hypothesized that the dogs were influenced by the owners’ unintentional cues. However, since humans typically show a stronger preference for rounded or curved shapes rather than spiky ones [51], this is unlikely. Another possible explanation could be that, in the case of dogs, animals’ facial configuration, especially when considering ancient dogs and wolves, is characterized by more angular, ‘spiky’ features, which contrasts with the round facial morphology typical of human infants and could plausibly contribute to an opposite bias. Finally, as we tested adult individuals, any early-life preference for roundness may no longer be sufficiently salient to influence their responses, which could instead be shaped by experiential factors (e.g. affordances for biting; the typical shape of food, treats or toys). It is also worth noting that in a study where dogs were required to generalize novel objects based on texture or shape during free exploration, their first approach was based on shape, but they later predominantly chose objects with similar texture [52]. Modifications in preference have also been observed in previous studies on cross-modal correspondences (e.g. space–luminance [14] and pitch–size [13]), where the strength of the association weakened over repeated trials, reaching chance level after about 20 repetitions. This is consistent with the ecological relevance of spontaneous associations in guiding initial organism–environment interactions. After repeated exposure or prolonged (visual) inspection, as in the case of the present study, it becomes more advantageous for the animal to adopt a different exploratory strategy to maximize the chances of finding something relevant. These hypotheses are not mutually exclusive, and one or several of them may help explain the reported effect; however, further empirical investigation is needed to directly assess their contribution.

Previous data on other primates failed to report the Bouba–Kiki effect on both great apes (gorillas and chimpanzees) [9] and bonobo [53], suggesting that it may be absent in non-human primates. If the Bouba–Kiki effect represents one instance of broader cross-modal correspondences, as supported by evidence from chicks and, based on our findings, also from dogs, it seems unlikely that primates would completely lack this form of cross-modal mapping. Indeed, primates show several other well documented cross-modal correspondences [11,12], indicating that the underlying mechanisms are present in this clade. Therefore, the failure to detect the Bouba–Kiki effect in these studies should be interpreted with caution, as it may reflect limitations related to the very small and heterogeneous samples tested, as well as procedural or contextual factors, rather than a true absence of the phenomenon.

If cross-modal correspondences evolved to prepare the individual for the kinds of associations they are probably to encounter in their environment, we can speculate that this ability would be shared across species that faced similar evolutionary pressures. Specifically, in the case of the Bouba–Kiki effect, it has been shown that this sound–shape correspondence mirrors the physical properties of sounds produced when spiky or round objects hit a surface [20]. As such, it is reasonable to predict that non-human species, including other mammals and birds [10], may respond to this association even in the absence of language. Nonetheless, dogs and humans constitute a special case because they have occupied a shared anthropogenic niche for approximately 32 000 years since dogs became the first domesticated species [54,55]. During this long co-evolutionary history, dogs have undergone substantial adaptations in neural, cognitive and communicative domains [21,56–62]. A study recently published [63] suggests that sound symbolism may facilitate effective interspecies communication between humans and dogs. The authors demonstrated that prosodic sound symbolism in human vocal commands may enhance mutual cross-species communication. Specifically, when interacting with dogs, owners show spontaneous congruent pitch–action associations when giving commands: the ‘down’ command has a significantly lower fundamental frequency than the ‘stand’ command [63]. It remains unclear whether this shared evolutionary history has led to greater similarities between humans and dogs, for example in the way predispositions for matching sounds to shape are exploited in communication. In humans, sound–shape associations are thought to provide a foundation for language emergence and vocabulary formation; it is still unknown whether dogs employ similar mechanisms when processing human speech or learning words and commands. Future research should investigate this topic more thoroughly, for example by conducting a direct comparison between domestic and wild animals to isolate the effect of co-evolution with humans, or by examining the role of sound symbolism in dogs’ perception and processing of human speech.

## 5. Conclusion

Our results provide evidence of the Bouba–Kiki effect in adult domestic dogs, contributing to recent literature that suggests this effect may represent a widespread case of sound–shape cross-modal correspondence. Further research should explore domestic dogs’ spontaneous preferences for different kinds of sounds and shapes, the presence of this association in early stages of life and the role of domestication and environment in favouring the emergence of sound–shape association.

## Supplementary Material



## Data Availability

All data underlying the analyses, statistical code (R scripts) and the two audio stimuli are publicly available on the Open Science Framework (OSF): [64]. Raw videos are not publicly available to protect the privacy of dog owners, but all derived data necessary to reproduce the analyses are available in the repository. Supplementary material is available online [65].
