# Supplementary material for: Hearing shapes: the Bouba–Kiki effect in adult domestic dogs
Source: Proc Biol Sci. 2026 Oct 14;293(2080):20261209. doi: 10.1098/rspb.2026.1209 (PMC13678620; doi:10.1098/rspb.2026.1209)
Supplement: Supplementary file 1 [file rspb.2026.1209.zip › rspb.2026.1209/rspb20261209_si_001.pdf]

Supplementary Materials for the Research Article – *Proceedings B Royal Society*

Hearing shapes: the Bouba-Kiki effect in adult domestic dogs

Eleonora Biffi[*1,7], Giulia Pedretti[2,7], Simona Normando[3], Teddy Lazebnik[4,5], Maria Loconsole[6], Paola Valsecchi[2].

1. University of Parma, Department of Medicine and Surgery, Via Gramsci 14, Parma, 43126, Italy
2. University of Parma, Department of Chemistry, Life Science and Environmental Sustainability, Viale delle Scienze 17/A, 43124, Parma, Italy
3. Comparative Biomedicine and Food Science Department, University of Padua, 35020 Legnaro, Italy.
4. Department of Information Systems, University of Haifa, Haifa, Israel.
5. Department of Computing, Jonkoping University, Jonkoping, Sweden.
6. Department of General Psychology, University of Padova, Padova, Italy.
7. Eleonora Biffi and Giulia Pedretti contributed equally to this work

**Corresponding author:* eleonora.biffi@unipr.it, *ORCID:* 0009-0006-3346-708X

Article DOI: 10.1098/rspb.2026.1209

Table S1.

Table of the dogs participated in the Study 1 (the owner was able to see the shapes and to hear the sounds) with the name, breed, age, sex, and the order of presentation of the stimuli during the test.

Subject	Age	Breed	Sex	Neutered	ORDER (8 possible)
Pepo	7	Bordercollie_mix	M	no	buba_kb; kiki_bk; buba_bk; kiki_kb
Quickly	9,5	Border_collie	M	yes	kiki_bk; buba_kb; kiki_kb; buba_bk
Macchia	10	Shepherd_mix	M	yes	buba_kb; kiki_bk; buba_bk; kiki_kb
Lea	12	Hunter_mix	F	yes	kiki_kb; buba_bk; kiki_bk; buba_kb
Reiki	2	Hunter_mix	F	yes	kiki_kb; buba_bk; kiki_bk; buba_kb
Mia	2	Poodle	F	no	kiki_kb; buba_kb; kiki_bk; buba_bk
Paco	7	Poodle	M	no	kiki_kb; buba_kb; kiki_bk; buba_bk
Luna	12	Labrador_mix	F	yes	kiki_bk; buba_bk; kiki_kb;buba_kb
Buddy	12	Labrador_mix	M	yes	kiki_bk; buba_bk; kiki_kb;buba_kb

Tokyo	1,5	Australian_shepherd	F	no	buba_bk; kiki_bk; buba_kb; kiki_kb
Pippo	11	Pinscher	M	no	kiki_kb; buba_kb; kiki_bk; buba_bk
Spritz	3	Shepherd_mix	M	no	buba_bk; kiki_kb; buba_kb; kiki_bk
Akemi	1,5	Border_collie	F	no	kiki_kb; buba_kb; kiki_bk; buba_bk
Jazz	4	Maremman_mix	M	yes	buba_bk; kiki_kb; buba_kb; kiki_bk
Dora	4,5	Hunter_mix	F	yes	kiki_kb; buba_bk; kiki_bk; buba_kb
Maya	10	Siberian_husky	F	yes	buba_kb; kiki_bk; buba_bk; kiki_kb
Attilia	5	Australian_shepherd	M	no	buba_bk; kiki_bk; buba_kb; kiki_kb
Diva	5	Belgian_malinois	F	yes	buba_kb; kiki_kb; buba_bk; kiki_bk
Malia	1,25	Belgian_malinois	F	no	kiki_bk; buba_kb; kiki_kb; buba_bk
Blu	11	Maremman_mix	M	yes	buba_bk; kiki_bk; buba_kb; kiki_kb
Max	5	German_shepherd	M	no	kiki_bk; buba_kb; kiki_kb; buba_bk
Astrid	4	German_shepherd	F	yes	kiki_kb; buba_bk; kiki_bk; buba_kb
Simba	2	Australian_shepherd	M	no	kiki_bk; buba_bk; kiki_kb;buba_kb
Diana	3	Beagle	F	yes	kiki_kb; buba_kb; kiki_bk; buba_bk
Luna	10	Cane_corso	F	yes	kiki_bk; buba_bk; kiki_kb;buba_kb
Eolo	8	Maltese	M	yes	buba_kb; kiki_kb; buba_bk; kiki_bk
Kaspijan	4	Dobermann	M	no	kiki_bk; buba_bk; kiki_kb;buba_kb

Thea	1,5	Lagorai_shepherd_mix	F	no	buba_kb; kiki_bk; buba_bk; kiki_kb
Oliver	11	Hunter_mix	M	no	buba_kb; kiki_kb; buba_bk; kiki_bk
Wendy	8	Golden_retriever	F	yes	buba_kb; kiki_kb; buba_bk; kiki_bk
Marley	10	Border_collie_mix	M	yes	kiki_bk; buba_kb; kiki_kb; buba_bk
Lady	11	Setter	F	yes	buba_bk; kiki_kb; buba_kb; kiki_bk
Samuel	8	Pointer	M	yes	buba_kb; kiki_bk; buba_bk; kiki_kb
Argo	7,5	Australian_shepherd	M	yes	buba_bk; kiki_bk; buba_kb; kiki_kb
Lola	8	German_shepherd	F	yes	buba_kb; kiki_kb; buba_bk; kiki_bk
Clint	10	Maremman_mix	M	no	buba_bk; kiki_bk; buba_kb; kiki_kb
Mission	6	Cane_lupo_italiano	F	yes	buba_kb; kiki_kb; buba_bk; kiki_bk
Gino	5	Bordercollie_mix	M	yes	buba_bk; kiki_kb; buba_kb; kiki_bk
Yago	2,5	Belgian_malinois	M	no	buba_bk; kiki_kb; buba_kb; kiki_bk
Alaska	6	Lupino_del_gigante	M	no	kiki_bk; buba_kb; kiki_kb; buba_bk
Cleo	11	Border_collie	F	yes	buba_kb; kiki_kb; buba_bk; kiki_bk
Luma	4	Bauceron	F	no	kiki_kb; buba_bk; kiki_bk; buba_kb
Buke	6	Bernese_mountain	M	no	kiki_kb; buba_kb; kiki_bk; buba_bk
Missy	2	Maltese	F	no	kiki_bk; buba_kb; kiki_kb; buba_bk
Amelie	1	Golden_retriever	F	no	kiki_kb; buba_bk; kiki_bk; buba_kb

Blues	4	Shepherd_mix	M	no	buba_kb; kiki_kb; buba_bk; kiki_bk
Argo	8	Hunter_mix	M	no	buba_bk; kiki_bk; buba_kb; kiki_kb
Olmo	5	Shepherd_mix	M	no	kiki_bk; buba_kb; kiki_kb; buba_bk
Gala	1	Border_collie	F	no	buba_bk; kiki_kb; buba_kb; kiki_bk
Chewbecca	4,5	Lagotto_romagnolo	M	no	buba_kb; kiki_bk; buba_bk; kiki_kb
Indie	12	Border_collie	F	yes	buba_bk; kiki_bk; buba_kb; kiki_kb

Table S2.

Table of the dogs participated in the Study 2 (with the owner wearing headphones playing music, but able to see the shapes) with the name, breed, age, sex, and the order of presentation of the stimuli during the test.

Subject	Age	Breed	Sex	Neutered	ORDER (8 possible)
Blues	3	Hunter_mix	M	no	buba_kb; kiki_bk; buba_bk; kiki_kb
Jack	4	Labrador_retriever	M	no	buba_bk; kiki_bk; buba_kb; kiki_kb
Pango	1,5	Labrador_retriever	M	no	buba_kb; kiki_kb; buba_bk; kiki_bk
Tilde	8	Lagorai_shepherd_mix	F	no	kiki_bk; buba_kb; kiki_kb; buba_bk
Yuma	4	Golden_retriever	F	no	kiki_kb; buba_bk; kiki_bk; buba_kb
Ondo	4	Dush_hund	M	no	kiki_bk; buba_bk; kiki_kb; buba_kb
Leo	11	Hunter_mix	M	yes	buba_kb; kiki_kb; buba_bk; kiki_bk
Luke	4	Shepherd_mix	M	yes	buba_bk; kiki_kb; buba_kb; kiki_bk
Milla	5,5	Lupino_gigante	F	no	buba_kb; kiki_bk; buba_bk; kiki_kb
Kylo	5	Shepherd_mix	F	yes	buba_bk; kiki_bk; buba_kb; kiki_kb
Mamy	8	Hunter_mix	F	yes	kiki_kb; buba_kb; kiki_bk; buba_bk
Yuki	3	Golden_retriever	F	no	kiki_bk; buba_kb; kiki_kb; buba_bk
Ambra	6,5	Italian_hound	F	yes	kiki_bk; buba_bk; kiki_kb; buba_kb
Guenda	6	Hunter_mix	F	yes	kiki_kb; buba_kb; kiki_bk; buba_bk
Sean	1,5	Australian_shepherd	M	no	buba_bk; kiki_kb; buba_kb; kiki_bk

Acli	5	Poodle	F	no	buba_kb; kiki_bk; buba_bk; kiki_kb
Nina	4	Hunter_mix	F	yes	buba_kb; kiki_kb; buba_bk; kiki_bk
Gino	5	Pinscher	M	no	buba_bk; kiki_bk; buba_kb; kiki_kb
Malva	1,5	Dachshund	F	yes	kiki_kb; buba_bk; kiki_bk; buba_kb

Table S3 – Eight possible combination order of auditory and position of the visual stimuli

**1**	BUBA_bk; KIKI_kb; BUBA_kb; KIKI_bk
**2**	BUBA_kb; KIKI_bk; BUBA_bk; KIKI_kb
**3**	BUBA_bk; KIKI_bk; BUBA_kb; KIKI_kb
**4**	BUBA_kb; KIKI_kb; BUBA_bk; KIKI_bk
**5**	KIKI_bk; BUBA_kb; KIKI_kb; BUBA_bk
**6**	KIKI_kb; BUBA_bk; KIKI_bk; BUBA_kb
**7**	KIKI_bk; BUBA_bk; KIKI_kb; BUBA_kb
**8**	KIKI_kb; BUBA_kb; KIKI_bk; BUBA_bk

Table S4.
List of behaviour coded manually for the exploration phase in the last 10 seconds of each trial.

Behaviour	Description	Behaviour type
Exploring bouba	Any anterior body part of the dog (nose, head, or front paws) entered the area delimited by the panel and the ground support corresponding to the screen on which the rounded shape was projected.	State event
Exploring kiki	Any anterior body part of the dog (nose, head, or front paws) entered the area delimited by the panel and the ground support corresponding to the screen on which the spiky shape was projected.	State event
No exploration	The dog did not enter either the rounded-shape area or the spiky-shape area.	State event

Table S5.
Analysis of time spent looking trial per trial in the Study 1 (the owner was able to see the shapes and to hear the sounds).

TRIAL	MEAN (sec)	RANGE (sec)	SD (sec)
1	5.44	1.132 – 11.851	2.24
2	5.84	0.001 – 11.167	2.32
3	5.99	0.962 – 9.311	2.01
4	6.09	1.749 – 8.905	1.97

Table S6.

Analysis of time spent looking trial per trial in Study 2 (the owner hearing headphones playing music, but able to see the shapes).

TRIAL	MEAN (sec)	RANGE (sec)	SD (sec)
1	8.79	6.336 – 11.969	1.85
2	9.57	7.017 – 11.787	1.44
3	8.27	6.085 – 11.853	1.68
4	8.73	6.055 – 11.861	1.94

Statistical models and results.

The models were the same for the two Studies.
The model stability was evaluated thanks to a function kindly provided by Roger Mundry ("diagnostics.plot"). The confidence interval, for all the models, was defined using "confint".

Model 1 - Creating the table of the looking time in which the mean, range, sd of looking at screen varies in the four trials

```
any(data_51$looking.on.screen <= 0)
data_51$looking.on.screen[data_51$looking.on.screen <= 0] = 0.001
risultati = data_51 %>%
  group_by(Trial) %>%
  summarise(
    mean_second_look = mean(looking.on.screen, na.rm = TRUE),
    range_second_look = paste0("[",
                        round(min(looking.on.screen, na.rm = TRUE), 3),
                        ", ",
                        round(max(looking.on.screen, na.rm = TRUE), 3),
                        "]"),
    sd_second_look = sd(looking.on.screen, na.rm = TRUE)
  )
```

Model 1.1 – Does the number of trial influence the time spent looking?

```
full_tot_look = glmmTMB(looking.on.screen ~ Trial + sex + age + (1 | Subject),
  family = Gamma(link = "log"),
  data = data)
null_tot_look = glmmTMB(looking.on.screen ~ Trial + sex + age + (1 | Subject),
  family = Gamma(link = "log"),
  data = data)
summary(full_tot_look)
```

Results Study 1

	Estimate	Std. Error	z-value	Pr(>|z|)
**(Intercept)**	1.657	0.105	15.793	<2e-16 ***
**Trial**	0.036	0.030	1.182	0.237
**sexM**	-0.024	0.068	-0.355	0.723
**age**	0.005	0.009	0.515	0.607

anova(full_tot_look, null_tot_look, test="Chisq")

	Df	AIC	BIC	logLik	deviance	X^2	Df	Pr(>Chisq)
**null**	5	976.12	992.72	-483.06	966.12			
**full**	6	976.73	996.64	-482.37	964.73	1.3915	1	0.2381

Results Study 2

	Estimate	Std. Error	z-value	Pr(>|z|)
**(Intercept)**	2.139	0.077	27.852	<2e-16
**Trial**	-0.016	0.02	-0.826	0.409
**sexM**	0.002	0.046	0.042	0.967
**age**	0.017	0.009	1.734	0.083

anova(full_tot_look, null_tot_look, test="Chisq")

	Df	AIC	BIC	logLik	deviance	X^2	Df	Pr(>Chisq)
**null**	5	305.69	317.34	-147.84	295.69			
**full**	6	307.00	320.99	-147.50	295.00	0.6794	1	0.4098

Model 2 – Do dogs have an overall looking preference toward one shape?

```
model.look.overall = glmmTMB(durata_looking ~ looking_at + Trial + sex + age + (1|Subject),
  family = beta_family(link = "logit"),
  data = data
)
null = glmmTMB(durata_looking ~  Trial + sex + age +(1|Subject),
  family = beta_family(link = "logit"),
  data = data
)
summary(model.look.overall)
```

Results Study 1

	Estimate	Std. Error	z-value	Pr(>|z|)	Lower CI	Upper CI
**(Intercept)**	-0.135	- 0.074	1.814	0.069	-0.280	-0.011
**looking_atspiky_shape_rate**	2.695e-01	0.045	5.928	< 0.001 ***	0.180	0.359
**Trial**	< -0.001	0.020	0.000	1.000	-0.040	0.040
**sexM**	< 0.001	0.046	0.000	1.000	-0.091	0.091
**age**	< 0.001	0.007	0.000	1.000	-0.013	0.013

anova(model.look.overall, null, test="Chisq")

	Df	AIC	BIC	logLik	deviance	X^2	Df	Pr(>Chisq)
**null**	6	-568.00	-543.94	290.00	-580.00			
**full**	7	-599.77	-571.69	306.88	-613.77	33.764	1	6.222e-09 ***

Results Study 2

	Estimate	Std. Error	z-value	Pr(>|z|)	Lower CI	Upper CI
**(Intercept)**	-0.026	0.147	-0.177	0.86	-0.313	0.262
**looking_atspiky_shape_rate**	0.052	0.083	0.628	0.530	-0.110	0.214
**Trial**	0.000	0.037	0.000	1.000	-0.072	0.072
**sexM**	< -0.0001	0.085	0.000	1.000	-0.167	0.167
**age**	< -0.0001	0.018	0.000	1.000	-0.035	0.035

anova(model_look_overall_s2, null_look_overall_s2, test = "Chisq")

	Df	AIC	BIC	logLik	deviance	X^2	Df	Pr(>Chisq)
**null**	6	-183.56	-165.42	97.781	-195.56			
**full**	7	-181.96	-160.79	97.978	-195.96	0.394	1	0.5302

Model 3 - Do dogs have a side biases towards right or left (independently to the sound played)?
Study 1 - Right:

```
t.test_R = t.test(side_bias_right$avg_right, mu = 0.5)
        One Sample t-test

data:  side_bias_right$avg_right
t = -0.24052, df = 50, p-value = 0.8109
alternative hypothesis: true mean is not equal to 0.5
95 percent confidence interval:
 0.4903435 0.5075911
sample estimates:
mean of x
0.4989673
```

Study 1 - Left:

```
t.test_L=t.test(side_bias_left$avg_left, mu = 0.5)
        One Sample t-test

data:  side_bias_left$avg_left
t = 0.24052, df = 50, p-value = 0.8109
alternative hypothesis: true mean is not equal to 0.5
95 percent confidence interval:
 0.4924089 0.5096565
sample estimates:
mean of x
0.5010327
```

Study 2 – Right:

```
t.test_R = t.test(side_bias_right$avg_right, mu = 0.5)
	One Sample t-test

data:  side_bias_right$avg_right
t = -0.32128, df = 18, p-value = 0.7517
alternative hypothesis: true mean is not equal to 0.5
95 percent confidence interval:
 0.4849428 0.5110628
sample estimates:
mean of x
0.4980028
```

Study 2 – Left:

```
t.test_L=t.test(side_bias_left$avg_left, mu = 0.5)
	One Sample t-test

data:  side_bias_left$avg_left
t = 0.32128, df = 18, p-value = 0.7517
alternative hypothesis: true mean is not equal to 0.5
95 percent confidence interval:
 0.4889372 0.5150572
sample estimates:
mean of x
0.5019972
```

Model 4 – Do dogs firstly look more toward one of the two shape?

Result study 1

```
chisq.test(table(first_look$shape))

	Chi-squared test for given probabilities

data:  table(first_look$shape)
X-squared = 1.9608, df = 1, p-value = 0.1614
```

Latency:

```
lat_full = glmmTMB(Latency ~ shape + (1|Subject),
  family =  Gamma(link = "log"),
  data = first_look)

lat_null = glmmTMB(Latency ~ (1|Subject),
  family =  Gamma(link = "log"),
  data = first_look)
```

	Estimate	Std. Error	z-value	Pr(>|z|)	Lower CI	Upper CI	min	max
**(Intercept)**	-2.174	0.058	-37.26	< 2e-16 ***	-2.289	-2.2058	-2.174	-2.197
**shapespiky**	-0.183	0.084	-2.18	0.0296 *	-0.349	-0.017	-0.183	-0.211

anova(lat_full, lat_null, test="Chisq")

	Df	AIC	BIC	logLik	deviance	X^2	Df	Pr(>Chisq)
**null**	3	-592.72	-582.76	299.36	-598.72			
**full**	4	-595.36	-582.09	301.68	-603.36	4.647	1	0.031 *

Results Study 2
chisq.test(table(first_look$shape))

Chi-squared test for given probabilities

data: table(first_look_2$shape)
X-squared = 0.47368, df = 1, p-value = 0.4913

Latency:

```
lat_full = glmmTMB(
  Latency ~ shape + (1|Subject),
  family =  Gamma(link = "log"),
  data = first_look_2)

lat_null = glmmTMB(
  Latency ~ (1|Subject),
  family =  Gamma(link = "log"),
  data = first_look_2)
```

	Estimate	Std. Error	z-value	Pr(>|z|)	Lower CI	Upper CI	min	max
**(Intercept)**	-2.383	0.100	-23.818	< 2e-16 ***	-2.575	-2.178	-2.435	-2.345
**shapespiky**	0.186	0.136	1.364	0.173	-0.086	0.455	0.115	0.265

anova(lat_full, lat_null, test="Chisq")

	Df	AIC	BIC	logLik	deviance	X^2	Df	Pr(>Chisq)
**null**	3	-222.56	-215.54	114.27	-228.53			
**full**	4	-222.36	-213.04	115.18	-230.36	1.828	1	0.176

Model 5 - Do dogs look more at the spiky shape when hearing the "Kiki" sound?

```
full_kiki = glmmTMB(spiky_shape_rate ~ Sound + Trial + sex + age + (1|Subject),
                    family = beta_family(link = "logit"),
                    data = data)
null_kiki = glmmTMB(spiky_shape_rate ~ Trial + sex + age + (1|Subject),
                    family = beta_family(link = "logit"),
                    data = data)
```

Results Study 1

	Estimate	Std. Error	z-value	Pr(>|z|)	Lower CI	Upper CI	min	max
**(Intercept)**	-0.183	0.066	-2.735	0.006 ***	-0.315	-0.051	-0.201	-0.159
**SoundKIKI**	0.764	0.037	20.410	< 2e-16 ***	0.689	0.837	0.749	0.780
**Trial**	-0.020	0.017	-1.250	0.211	-0.054	0.012	-0.026	-0.015
**sexM**	-0.047	0.047	-1.010	0.312	-0.140	0.046	-0.063	-0.024
**age**	0.002	0.006	0.366	0.714	-0.010	0.015	-0.001	0.004

anova(full_kiki, null_kiki, test="Chisq")

	Df	AIC	BIC	logLik	deviance	X^2	Df	Pr(>Chisq)
**null**	6	-295.62	-275.71	153.81	-307.62			
**full**	7	-509.31	-486.09	261.66	-523.31	215.69	1	< 2.2e-16 ***

Results Study 2

	Estimate	Std. Error	z-value	Pr(>|z|)	Lower CI	Upper CI	min	max
**(Intercept)**	-0.572	0.095	-5.997	2.01e-09 ***	-0.764	-0.382	-0.653	-0.524
**SoundKIKI**	0.972	0.048	20.242	< 2e-16 ***	0.876	1.068	0.959	0.991
**age**	0.012	0.012	1.036	0.300	-0.012	0.037	0.008	0.028
**sexM**	0.025	0.057	0.437	0.662	-0.093	0.144	0.000	0.067
**OrderKB**	-0.015	0.048	-0.320	0.749	-0.111	0.080	-0.041	0.009
**Trial**	0.020	0.021	0.947	0.344	-0.023	0.063	0.009	0.031

anova(full_kiki, null_kiki, test="Chisq")

	Df	AIC	BIC	logLik	deviance	X^2	Df	Pr(>Chisq)
**null**	7	-85.076	-68.761	49.428	-99.076			
**full**	8	-220.494	-201.848	118.247	-236.494	137.42	1	< 2.2e-16 ***

Model 6 - Do dogs look more at the round shape when hearing the "Bouba" sound?

```
full_bouba = glmmTMB(rounded_shape_rate ~ Sound + Trial + sex + age + (1|Subject),
        family = beta_family(link = "logit"),
        data = data)
null_bouba = glmmTMB(rounded_shape_rate ~ Trial + sex + age + (1|Subject),
        family = beta_family(link = "logit"),
        data = data)
```

Results Study 1

	Estimate	Std. Error	z-value	Pr(>|z|)	Lower CI	Upper CI	min	max
**(Intercept)**	0.183	0.066	2.735	0.006 ***	0.051	0.315	0.159	0.201
**soundKIKI**	-0.764	0.037	-20.410	< 2e-16 ***	-0.837	-0.689	-0.780	-0.749
**Trial**	0.020	0.017	1.250	0.211	-0.012	0.054	0.015	0.026
**sexM**	0.047	0.047	1.010	0.312	-0.046	0.140	0.024	0.063
**age**	-0.002	0.006	-0.366	0.714	-0.015	0.010	-0.004	-0.001

anova(full_bouba, null_bouba, test="Chisq")

	Df	AIC	BIC	logLik	deviance	X^2	Df	Pr(>Chisq)
**null**	6	-295.62	-275.71	153.81	-307.62			
**full**	7	-509.31	-486.09	261.66	-523.31	215.69	1	< 2.2e-16 ***

Results Study 2

	Estimate	Std. Error	z-value	Pr(>|z|)	Lower CI	Upper CI	min	max
**(Intercept)**	0.572	0.095	5.997	2.01e-09 ***	0.382	0.764	0.524	0.653
**SoundKIKI**	-0.972	0.048	-20.242	< 2e-16 ***	-1.068	-0.876	-0.991	-0.959
**OrderKB**	0.015	0.048	0.320	0.749	-0.080	0.111	-0.009	0.041
**sexM**	-0.025	0.057	-0.437	0.662	-0.144	0.093	-0.067	0.000
**age**	-0.012	0.012	1.036	0.300	-0.037	0.012	-0.028	-0.008
**Trial**	-0.020	0.021	-0.947	0.344	-0.063	0.022	-0.031	-0.009

anova(full_bouba, null_bouba, test="Chisq")

	Df	AIC	BIC	logLik	deviance	X^2	Df	Pr(>Chisq)
**null**	7	-85.976	-68.761	49.538	-99.076			
**full**	8	-220.494	-201.848	118.247	-236.494	137.85	1	< 2.2e-16 ***

Model 7 – Exploration phase

7.1.1 - Do dogs explore more the round shape when hearing the "Bouba" sound (occurrence)?

```
exp_buba = subset(output, Behavior == "exp_buba")
full_buba_binomial_exp = glmmTMB(yes_no_exp ~ trial + sound + sex + age + (1|subject),
                       family = binomial(link = "logit"),
                       data = exp_buba)
```

```
null_buba_binomial_exp = glmmTMB(yes_no_exp ~ trial + sex + age + (1|subject),
                                family = binomial(link = "logit"),
                                data = exp_buba)
```

Results Study 1

	Estimate	Std. Error	z-value	Pr(>|z|)	Lower CI	Upper CI	min	max
**(Intercept)**	-1.042	0.577	-1.807	0.070	-2-234	0.063	-1.273	-0.853
**Trial**	-0.056	0.152	-0.365	0.715	-0.358	0.243	-0.107	-0.010
**SoundKIKI**	-0.170	0.341	-0.499	0.618	-0.847	0.498	-0.237	-0.052
**sexM**	0.012	0.378	0.033	0.974	-0.763	0.781	-0.182	0.147
**age**	-0.003	0.053	-0.062	0.951	-0.113	0.104	-0.020	0.015

anova(full_buba_binomial_exp, null_buba_binomial_exp, test="Chisq")

	Df	AIC	BIC	logLik	deviance	X^2	Df	Pr(>Chisq)
**null**	5	229.42	246.01	-109.71	219.42			
**full**	6	231.18	251.08	-109.59	219.18	0.249	1	0.6175

Results Study 2

	Estimate	Std. Error	z-value	Pr(>|z|)	Lower CI	Upper CI	min	max
**(Intercept)**	-3.996	3.903	-1.024	0.306	-16.830	2.343	-10.412	-2.259
**SoundKIKI**	-1.143	1.210	-0.944	0.345	-4.212	0.974	-21.834	-0.667
**Trial**	0.209	0.492	0.426	0.670	-0.763	1.397	-0.153	1.071
**age**	-0.024	0.425	-0.056	0.955	-1.616	1.115	-0.172	0.450
**sexM**	-1.487	2.017	-0.737	0.461	-10.507	4.461	-22.264	-0.510

anova(full_buba_binomial_exp, null_buba_binomial_exp, test="Chisq")

	Df	AIC	BIC	logLik	deviance	X^2	Df	Pr(>Chisq)
**null**	5	46.041	57.694	-18.020	36.041			
**full**	6	46.979	60.963	-17.489	34.979	1.062	1	0.3028

7.1.2 Do dogs explore more the round shape when hearing the "Bouba" sound (duration)?

```
full_buba_dur = glmmTMB(Duration ~  sound + trial + sex + age + (1|subject),
                       family = Gamma(link = "log"),
                       data = exp_buba)
null_buba_dur = glmmTMB(Duration ~  trial + sex + age + (1|subject),
                       family = Gamma(link = "log"),
```

data = exp_buba)

Results Study 1

	Estimate	Std. Error	z-value	Pr(>|z|)	Lower CI	Upper CI	min	max
**(Intercept)**	1.017	0.419	2.423	0.015	0.208	1.883	0.644	1.187
**SoundKIKI**	0.505	0.276	1.829	0.067	-0.050	1.055	0.352	0.596
**Trial**	-0.043	0.112	-0.383	0.701	-0.266	0.185	-0.078	0.015
**sexM**	-0.195	0.269	-0.722	0.470	-0.739	0.341	-0.320	-0.105
**age**	-0.054	0.042	-1.302	0.193	-0.136	0.031	-0.079	-0.031

anova(full_buba_dur, null_buba_dur, test="Chisq")

	Df	AIC	BIC	logLik	deviance	X^2	Df	Pr(>Chisq)
**null**	6	173.33	184.43	-80.664	161.33			
**full**	7	172.13	185.08	-79.064	158.13	3.2009	1	0.736

Results Study 2

	Estimate	Std. Error	z-value	Pr(>|z|)	Lower CI	Upper CI	min	max
**(Intercept)**	-5.668	2.458	-2.305	0.021*	-10.731	-0.626	-7.219	-4.603
**SoundKIKI**	-0.464	0.415	-1.120	0.263	-1.333	0.335	-0.616	-0.272
**Trial**	0.050	0.177	0.284	0.776	-0302	0.406	-0.050	0.142
**age**	-0.05	0.404	-0.131	0.896	-0.886	0.781	-0.190	0.235
**sexM**	-1.653	1.935	-0.855	0.393	-5.638	2.339	-2.742	-0.859

anova(full_buba_dur, null_buba_dur, test="Chisq")

	Df	AIC	BIC	logLik	deviance	X^2	Df	Pr(>Chisq)
**null**	6	-852.49	-838.51	432.25	-864.49			
**full**	7	-851.78	-835.47	432.89	-865.78	1.2924	1	0.2556

7.2 Do dogs explore more the spiky shape when hearing the "Kiki" sound (occurrence)?

```
exp_kiki= subset(output, Behavior == "exp_kiki")
full_kiki_binomial_exp = glmmTMB(yes_no_exp ~  sound + trial + sex + age + (1|subject),
                         family = binomial(link = "logit"),
                          data = exp_kiki)
null_kiki_binomial_exp = glmmTMB(yes_no_exp ~ trial + sex + age + (1|subject),
                          family = binomial(link = "logit"),
```

data = exp_kiki)

Results Study 1

	Estimate	Std. Error	z-value	Pr(>|z|)	Lower CI	Upper CI	min	max
**(Intercept)**	-2.230	0.694	-3.208	0.001 **	-3.731	-0.950	-2.530	-2.097
**SoundKIKI**	1.069	0.400	2.671	0.007 **	0.311	1.895	0.959	1.207
**Trial**	-0.034	0.169	-0.199	0.842	-0.369	0.299	-0.092	0.015
**sexM**	0.518	0.440	1.176	0.240	-0.356	1.454	0.346	0.686
**age**	-0.027	0.062	-0.435	0.664	-0.156	0.099	-0.051	-0.003

anova(full_ kiki_binomial_exp null_ kiki_binomial_exp, test="Chisq")

	Df	AIC	BIC	logLik	deviance	X^2	Df	Pr(>Chisq)
**null**	5	206.68	223.27	-98.340	196.68			
**full**	6	200.88	220.79	-94.441	188.88	7.797	1	0.005 ***

Results Study 2

	Estimate	Std. Error	z-value	Pr(>|z|)	Lower CI	Upper CI	min	max
**(Intercept)**	0.841	1.596	0.526	0.599	-2.306	4.089	0.010	2.256
**SoundKIKI**	-0.411	0.856	-0.480	0.631	-2.197	1.270	-0.949	0.022
**Trial**	-0.672	0.419	-1.606	0.108	-1.600	0.093	-0.972	-0.461
**age**	-0.515	0.280	-1.841	0.066	-1.134	-0.052	-0.735	-0.423
**sexM**	1.149	0.927	1.240	0.215	-0.578	3.222	0.414	1.793

anova(full_ kiki_binomial_exp, null_ kiki_binomial_exp, test="Chisq")

	Df	AIC	BIC	logLik	deviance	X^2	Df	Pr(>Chisq)
**null**	5	49.127	60.781	-19.564	39.127			
**full**	6	50.894	64.879	-19.447	38.894	0.2328	1	0.6294

7.2.1 – Do dogs explore more the spiky shape when hearing the "Kiki" sound (duration)?

```
full_kiki_dur = glmmTMB(Duration_exp ~  sound + trial + sex + age + (1|subject),
                        family = Gamma(link = "log"),
                        data = exp_kiki )
null_kiki_dur = glmmTMB(Duration_exp ~  trial + sex + age + (1|subject),
                   family = Gamma(link = "log"),
                   data = exp_kiki )
```

Results Study 1

	Estimate	Std. Error	z-value	Pr(>|z|)	Lower CI	Upper CI	min	max
**(Intercept)**	-5.314	1.279	-4.153	3.28e-05 ***	-7.848	-2.761	-5.906	-4.940
**SoundKIKI**	1.972	0.605	3.259	0.001 **	0.891	3.289	1.690	2.272
**Trial**	-0.228	0.204	-1.114	0.265	-0.645	0.166	-0.333	-0.134
**sexM**	1.329	1.099	1.209	0.227	-0.888	3.504	1.075	0.068
**age**	0.027	0.154	0.173	0.863	-0.281	0.336	-0.022	0.068

anova(full_tot_look, null_tot_look, test="Chisq")

	Df	AIC	BIC	logLik	deviance	X^2	Df	Pr(>Chisq)
**null**	6	-1748.5	-1728.6	880.23	-1760.5			
**full**	7	-1760.9	-1737.7	887.44	-1774.9	14.406	1	0.0001***

Results Study 2

	Estimate	Std. Error	z-value	Pr(>|z|)	Lower CI	Upper CI	min	max
**(Intercept)**	-1.1707	2.312	-0.506	0.613	-5.977	3.488	-2.863	0.673
**SoundKIKI**	-0.347	0.523	-0.663	0.507	-1.440	0.678	-0.582	-0.135
**Trial**	-0.349	0.263	-1.324	0.185	-0.918	0.151	-0.391	-0.212
**age**	-0.904	0.373	-2.424	0.02	-1.673	-0.132	-1.129	-0.765
**sexM**	2.488	1.788	1.392	0.164	-1.200	6.175	1.707	3.337

anova(full_kiki_duration, null_kiki_duration, test="Chisq")

	Df	AIC	BIC	logLik	deviance	X^2	Df	Pr(>Chisq)
**null**	6	-770.95	-756.96	391.47	-782.95			
**full**	7	-769.39	-753.08	391.70	-783.39	0.4468	1	0.5039
